# Bis-Pyridine-Based Organogel with AIE Effect and Sensing Performance towards Hg^2+^

**DOI:** 10.3390/gels8080464

**Published:** 2022-07-25

**Authors:** Aiping Gao, Qingqing Han, Qingqing Wang, Rong Wan, Huijuan Wu, Xinhua Cao

**Affiliations:** College of Chemistry and Chemical Engineering and Synthesis Key Laboratory of Xinyang City, Xinyang Normal University, Xinyang 464000, China; gaoapchem@163.com (A.G.); hqq9627@163.com (Q.H.); 18939697579@163.com (Q.W.); wuhjchem@163.com (H.W.)

**Keywords:** bis-pyridine, self-assembly, supramolecular gel, detection of Hg^2+^

## Abstract

A novel gelator (**1**) based on a bis-pyridine derivative was designed and synthesized, which could form stable gels in methanol, ethanol, acetonitrile, ethyl acetate, DMF/H_2_O (4/1, *v*/*v*) and DMSO/H_2_O (4/1, *v*/*v*). The self-assembly process of gelator **1** was studied by field emission scanning electron microscopy (FESEM), UV–vis absorption spectroscopy, fluorescence emission spectroscopy, Fourier transform infrared spectroscopy (FT-IR), X-ray powder diffraction and a water contact angle experiment. Gelator **1** exhibited obvious AIE behavior. On the base of its AIE, the gel of **1** could detect Hg^2+^, which resulted in fluorescence quenching and a gel–sol transition. ^1^H NMR titration experiments with Hg^2+^ revealed that the metal coordination interaction induced the fluorescence quenching and the breakdown of the noncovalent interaction in the gel system. This research provides a new molecular mode for designing a functional self-assembly gel system.

## 1. Introduction 

In the past thirty years, supramolecular chemistry has become even more sophisticated and quickly developed due to its applications in a variety of disciplines and fields [1,2,3,4,5,6,7,8,9,10,11]. As a typical supramolecular material, low-molecular-weight gels (LMWGs) are a kind of soft material between a solid and liquid which have been developed rapidly and widely used in electrolyte materials [12], sensors [13,14], drug delivery systems [15], liquid crystal [16], supramolecular chirality [17,18,19], light-harvesting systems [20,21], surfactants [22] and other fields [23,24,25,26,27,28]. Organic molecules can be self-assembled into organogel or hydrogel under the driving force of noncovalent interactions, such as a hydrogen bond, metal coordination, π–π stacking, hydrophobic effect, electrostatic force and van der Waals force [29,30,31,32,33]. Due to the weakness and reversibility of the aforementioned noncovalent interactions, LMWGs usually can respond to environment stimuli including light, pH, temperature, mechanical stress and chemical stimuli and lead to a change in color, emission or physical state [34,35,36,37,38]. If supramolecular gels are used as a sensor, they can exhibit unique superiority due to the three-dimensional network and nature of noncovalent interaction. For example, Wei et al. constructed a new stimuli-responsive supramolecular polymer system with the capability of ultrasensitive detection and separation of Pb^2+^, Cr^3+^ and Fe^3+^ via the competitive coordination effect [39]. We also designed several self-assembly gel systems with the ability to detect multianalytes [40,41,42].

Mercury is one of the heavy metals and is extremely toxic to the human body and environment [43,44,45]. The accumulation of mercury ions in the water environment pollutes aquatic animals and plants and threatens their survival and health [46]. Mercury enriched in fish and shrimp may enter the human body through the food chain and cause diseases including brain damage and chronic disease [47,48]. Many methods and materials have been used for the detection of mercury ions [49,50,51,52,53,54]. Therefore, there is a great demand to develop a simple, rapid and highly selective sensor for mercury ions. 

**Scheme 1:**
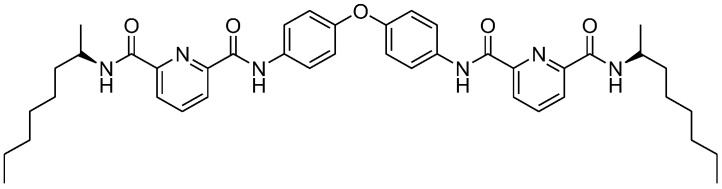
The structure of gelator **1**.

In this work, a novel bis-pyridine gelator (**1**) could form gels in some pure solvents and mixed solvents (Scheme 1). Gelator **1** was originally designed for the construction of a metal-coordinated, tunable supramolecular chiral self-assembly system via the introduction of two pyridines and (R)-octan-2-amine. Unfortunately, supramolecular chirality did not present in the self-assembly systems, and the metal coordination effect did not induce and adjust supramolecular chirality in the gels of **1**. AIE behavior widely exists in many fields including MOFs, polymers, etc. [55,56,57,58,59]. It was meaningful that gels of **1** exhibited an obvious AIE property in the sol–gel transition and a good response ability towards mercury ions. The self-assembly processes of **1** in different solvents and the detection of mercury ions by the gel of **1** were investigated in detail. This work could bring a new perspective to the designing of functional supramolecular gels as fluorescent sensors. 

## 2. Results and Discussion

**Figure 1:**
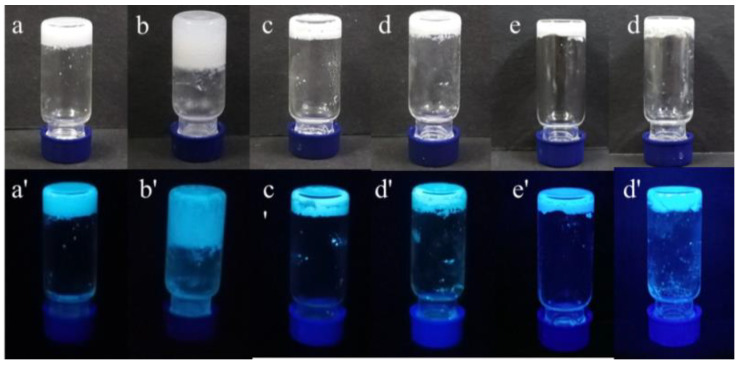
Images of gels of **1** in different solvents: (**a**,**a′**) DMF/H_2_O (4/1, *v*/*v*); (**b**,**b′**) DMSO/H_2_O (4/1, *v*/*v*); (**c**,**c′**) methanol; (**d**,**d′**) ethanol; (**e**,**e′**) ethyl acetate; (**f**,**f′**) acetonitrile (gel concentration is its corresponding CGC).

A new gelator based on a bis-pyridine derivative was designed and synthesized, and the detailed characterization data are shown in the supporting information (SI). The gelation performance of **1** was explored in a series of solvents ranging from nonpolar to polar via the method of inverted tubes, and the results are shown in Table 1 [60]. Gelator **1** could form white gel in methanol, ethanol, acetonitrile, 1, 4-dioxane, ethyl acetate, DMSO/H_2_O (4:1, *v*/*v*) and DMF/H_2_O (4:1, *v*/*v*) with the critical gel concentration (CGC) of 25, 25, 25, 8.33, 25, 6.67 and 20 mg mL^−1^, respectively. Precipitate **1** was observed in toluene and 1, 4-dioxane. In n-hexane, petroleum ether and acetone, compound **1** was not completely dissolved at the concentration of 25 mg mL^−1^. On the contrary, solution **1** was obtained in THF. The gels of **1** in these solvents could be stable for several months. In addition, these gels possessed obvious AIE behavior, and the nonfluorescent solution was changed to fluorescent gel in the sol–gel transition process. The gels of **1** emitted blue light under 365 nm light (Figure 1).

**Figure 2:**
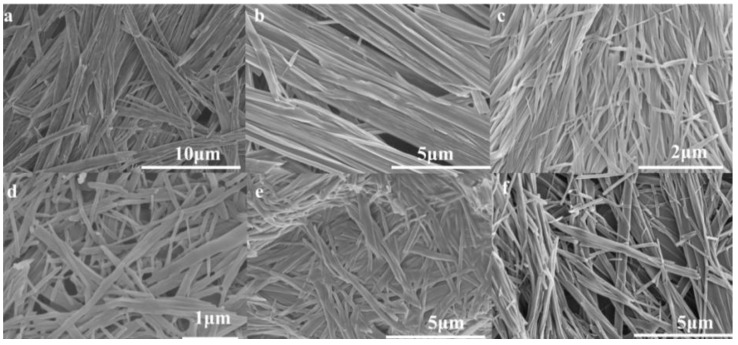
SEM images of xerogels of **1** in (**a**) DMF/H_2_O (4/1, *v*/*v*), (**b**) acetonitrile, (**c**) ethyl acetate, (**d**) DMSO/H_2_O (4/1, *v*/*v*), (**e**) ethanol, (**f**) methanol.

To observe the self-assembly morphologies in progress, FESEM was performed on the xerogels of **1** in DMSO/H_2_O (4/1, *v*/*v*), DMF/H_2_O (4/1, *v*/*v*), methanol, ethanol, acetonitrile and ethyl acetate to reveal their self-assembly structures (Figure 2). The diluted gel of **1** was spread evenly on mica, and dried by freeze-drying technology. As shown in Figure 2a, gelator **1** self-assembled into irregular microbelts with the length of several micrometers and the width of 0–2 μm, and then stacked into three-dimensional structures in DMF. Similar structures were observed in other xerogels of **1**. There was a regular arrangement in the xerogels of **1** in acetonitrile and ethyl acetate with different interspaces in their three-dimensional structures (Figure 2b,c). The structure of the gel of **1** in DMSO/H_2_O (4/1, *v*/*v*) demonstrated chaotic microbelts with a width of about 0.2–0.5 micrometers and a length of several micrometers (Figure 2d). It could be observed that dense microbelts intertwined with the three-dimensional network in the gel of **1** in ethanol in Figure 2e. The most porous three-dimensional network constructed by irregular nanorods was found in the gel of **1** in methanol (Figure 2f). The above results showed that the solvent had a certain effect on the self-assembly of gelator **1** through the interaction between the gelator molecule and solvent molecule.

**Figure 3:**
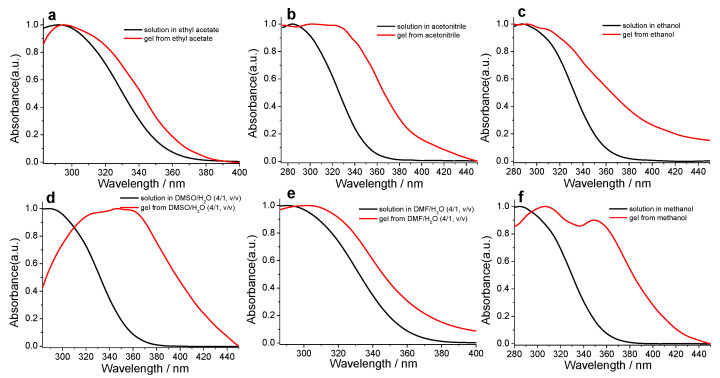
UV–vis absorption spectra of gelator **1** in solution and gel state with different solvents: (**a**) ethyl acetate; (**b**) acetonitrile; (**c**) ethanol; (**d**) DMSO/H_2_O (4/1, *v*/*v*); (**e**) DMF/H_2_O (4/1, *v*/*v*); (**f**) methanol. The concentrations of solution and gel were 10^−5^ M and the corresponding CGC.

The molecular self-assembly pattern in the gel system can be explored by comparing the UV–vis absorption spectra of the solution and gel state. UV–vis absorption spectra of gelator **1** in a solution and gel state with different solvents were obtained and shown in Figure 3. The maximum absorption band at 292 nm of gelator **1** in ethyl acetate was slightly red-shifted to 295 nm in its gel state (Figure 3a). Similar phenomena also appeared in other UV–vis absorption spectra of gelator **1** in other solvents. In acetonitrile, the maximum absorption band of 284 nm of solution **1** was moved to 301 nm with the red-shift of 17 nm for the gel state (Figure 3b). The UV–vis absorption band of solution **1** in ethanol was at 288 nm, which had just a red-shift of 3 nm for its gel state (Figure 3c). Solution **1** in DMSO/H_2_O (4/1, *v*/*v*) had an absorption band at 288 nm, which was changed into a broad band at 347 nm for its gel state, indicating the J-aggregation self-assembly mode existed in the gel system [61] (Figure 3d). The maximum absorption band of solution **1** in DMF was 290 nm and red-shifted to 302 nm for its gel state (Figure 3e). It was different for solution **1** in methanol, which had an absorption band at 285 nm that was changed into two bands at 306 nm and 349 nm. The slow decay in absorbance in Figure 3c,e was possible due to the scattering effect of the gels.

**Figure 4:**
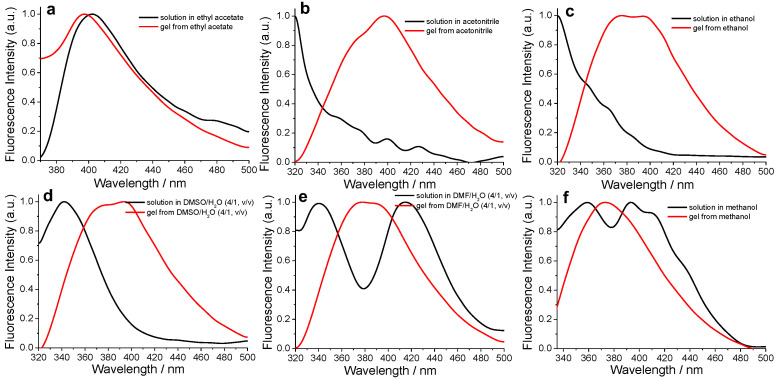
Fluorescence emission spectra of **1** in solution and gel state with different solvents. The concentration of solution was 10^−5^ M and CGC. (**a**) Ethyl acetate; (**b**) acetonitrile; (**c**) ethanol; (**d**) DMSO/H_2_O (4/1, *v*/*v*); (**e**) DMF/H_2_O (4/1, *v*/*v*); (**f**) methanol.

To explore the effect of solvents on gelator **1**, fluorescence emission spectra of **1** in solution and gel states with six different solvents were investigated (Figure 4). In Figure 4a, solution **1** in ethyl acetate had a maximum emission peak at 402 nm with a very weak intensity, and was blue-shifted to 397 nm in the gel state, indicating the TICT behavior of gelator **1** in ethyl acetate [62]. Solution **1** in acetonitrile almost did not emit light, and an emission peak was at 393 nm for its gel state (Figure 4b). Similar emission behavior was observed for the gel of **1** in ethanol (Figure 4c). An emission at 342 nm of solution **1** in DMSO/H_2_O (4/1, *v*/*v*) was red-shifted to 393 nm with a shoulder peak of 376 nm in the gel state, which showed that π–π stacking was presented in the gel system. Two emission peaks appeared at 339 and 415 nm for solution **1** in DMF/H_2_O (4/1, *v*/*v*), which combined into a single broad peak at 378 nm in the gel state (Figure 4e). Two emission peaks at 358 and 393 nm of solution **1** in methanol were also changed into one peak at 373 nm in the gel state (Figure 4f). These experimental results exhibited that the solvent not only affected the self-assembly process, but also influenced the emissions.

An X-ray powder diffraction experiment can provide some information about the self-assembly patterns in gel [63]. Xerogels of **1** were explored by the XRD experiment (Figure S1). As shown in Figure S1a,a′, the XRD pattern of the xerogel of **1** in acetonitrile showed a series of diffraction peaks with the *d*-space values of 2.69, 1.35, 0.84, 0.76, 0.67, 0.59, 0.56, 0.50, 0.45, 0.43, 0.40, 0.36, 0.34 and 0.31 nm. The *d*-space values of 2.69, 1.35 and 0.67 nm were at the ratio of 1: 1/2: 1/4 and indicated that gelator **1** was self-assembled into a layered structure with a layer distance of 2.69 nm, which was close to the molecular length of gelator **1** and showed the monomolecular self-assembly. The similar diffraction peaks and *d*-space values were also presented in other XRD patterns of the xerogels of **1**, indicating that the self-assembly of gelator **1** in gel systems was highly ordered (Figure S1b–f,b′–f′). The XRD pattern of the powder of **1** showed only one diffraction peak at 2θ = 20°, which indicated that molecule **1** in powder was not in orderly arrangement such as that of the gel state (Figure S1g,g′).

**Figure 5:**
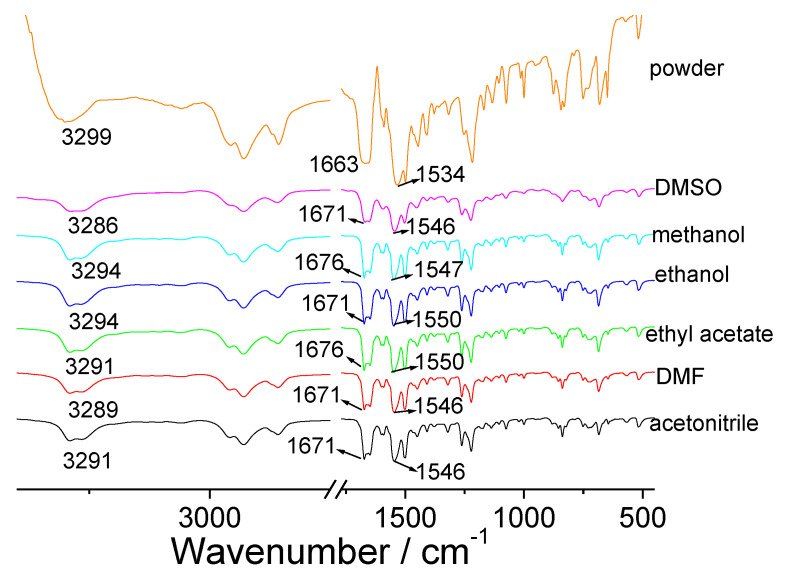
FT IR spectra of xerogels of **1** in different solvents and powder **1**.

It is well known that there are one or more driving forces, such as a hydrogen bond and van der Waals force, in the self-assembly gel [64]. In order to analyze hydrogen bonding in the gels of **1** in different solvents, Fourier transform infrared spectra of xerogels of **1** were obtained. As shown in Figure 5, N–H stretching vibration peaks of xerogels of **1** in methanol, ethanol, ethyl acetate, DMSO/H_2_O (4/1, *v*/*v*), DMF/H_2_O (4/1, *v*/*v*) and acetonitrile were at 3294, 3294, 3291, 3286, 3259 and 3291 cm^−1^, respectively. Furthermore, the stretching vibration peaks of C=O (amide I and II) of xerogels of **1** in the aforementioned solvents and powder **1** were at 1676, 1671, 1676, 1671, 1671 and 1671 cm^−1^ and 1546, 1547, 1550, 1546, 1546 and 1546 cm^−1^, respectively. The results demonstrated that there were intermolecular hydrogen bonds in the gel system. The FTIR spectrum of powder **1** showed that N–H and C=O (amide I and II) stretching vibration peaks were at 3299, 1663 and 1534 cm^−1^, which demonstrated that a hydrogen bond also existed in the powder state.

**Figure 6:**
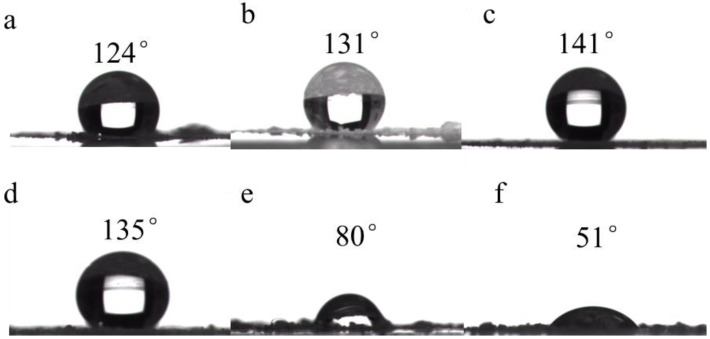
Imaging of water contact angle of xerogels of **1** coated with different solvents. (**a**) DMF/H_2_O (4/1, *v*/*v*); (**b**) acetonitrile; (**c**) DMSO/H_2_O (4/1, *v*/*v*); (**d**) ethyl acetate; (**e**) ethanol; (**f**) methanol.

Surface wettability plays an important role in material application, especially as superhydrophobic and superhydrophilic surfaces have many potential applications [65,66,67]. The diluted gel of **1** was spread on the glass sheet with a certain thickness to avoid the basal influence, and then dried naturally. As shown in Figure 6a–d, xerogels of **1** in DMF/H_2_O (4/1, *v*/*v*), acetonitrile, DMSO/H_2_O (4/1, *v*/*v*) and ethyl acetate were hydrophobic with water contact angles of 124°, 131°, 141° and 135°, respectively, whereas xerogels of **1** formed in ethanol and methanol exhibited superhydrophilicity with small contact angles of 80° and 51° (Figure 6e,f). For the above experimental results, it was concluded that the surface wettability of xerogels of **1** was mainly determined by their different self-assembly modes and structures due to their same components.

**Figure 7:**
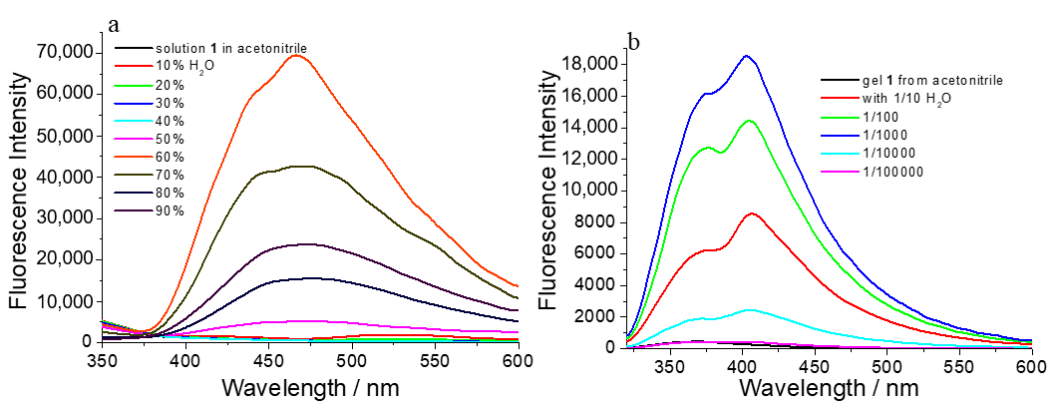
Fluorescence emission spectra of solution (10^−5^ M) (**a**) and gel of **1** (CGC) (**b**) in acetonitrile with different water content.

To further verify the AIE of gelator **1**, the fluorescence emissions of gelator **1** in the solution and gel state with different water proportions were obtained (Figure 7). With water content up to 70%, the emission of solution **1** in acetonitrile was greatly enhanced by 75 times, and then was decreased after the water content was more than 70% (Figure 7a). The fluorescence emission change of solution **1** in acetonitrile with different contents of water was well verified by the corresponding images (Figure S2). It was regrettable that the emission light from solution **1** with 70% water was not observed under a portable UV lamp. A similar emission change was observed in the gel system (Figure 7b). When the water proportion increased from 1/100,000 to 1/1000, the fluorescence intensity of the gel of **1** also continuously increased. If the water proportion was more than 1/1000, the fluorescence intensity was gradually decreased. However, the overall fluorescence intensity was higher than that of the gel formed in pure acetonitrile. Two emission peaks appeared at 373 nm and 403 nm after the water content increased to 1/10,000. 

**Figure 8:**
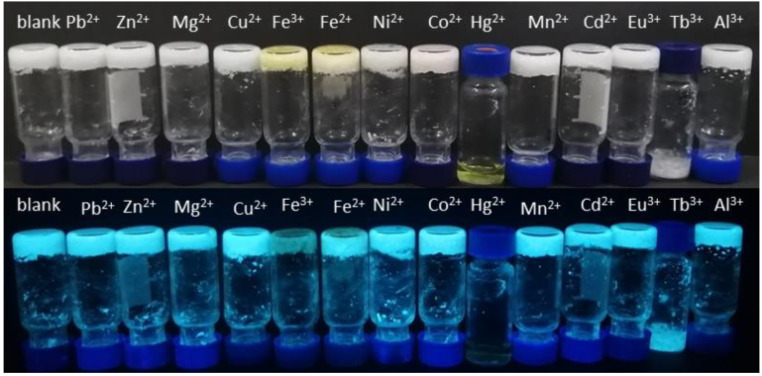
The images of gels of **1** with different metal ions.

**Figure 9:**
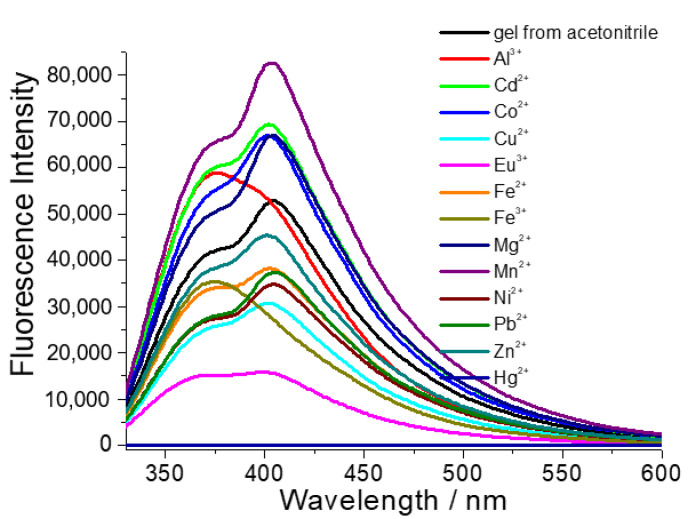
Fluorescence emission spectra of gels of **1** with different metal ions (1.0 eq.).

Based on the coordination interaction of the pyridine group in molecule **1** and its AIE effect in a gel state, we tried to explore the responsive ability of the gel of **1** to different metal ions [68,69,70,71]. Fourteen common metal ions were selected in this experiment. First, 1.0 eq. of metal ions was added to the gel of **1** in acetonitrile, and then the gel was melted and reforged in the presence of the ions for inspection of the responsive ability via the change in the fluorescence and gel state. In Figure 8, the state and color of the gel of **1** did not exhibit significant change after the addition of Al^3+^, Mg^2+^, Mn^2+^, Cd^2+^, Co^2+^, Cu^2+^, Ni^2+^, Pb^2+^, Eu^3+^ and Zn^2+^. The gel state did not change after the addition of Fe^2+^ and Fe^3+^, but the color changed to light yellow. Under 365 nm light irradiation, the emission of the gel of **1** with Fe^3+^ decreased. The gel containing Hg^2+^ exhibited the most obvious change. The white gel was collapsed into a pale yellow solution along with the disappearance of its fluorescence. The addition of Tb^3+^ also induced the collapse of the gel, but the fluorescence was still kept. To better observe the fluorescence change with 1.0 eq. ions, the fluorescence emissions of gels of **1** with different ions were obtained (Figure 9). The addition of 1.0 eq. of Al^3+^, Cd^2+^, Co^2+^, Cu^2+^, Fe^2+^, Fe^3+^, Mg^2+^, Mn^2+^, Ni^2+^, Pb^2+^ and Zn^2+^ all induced the fluctuations in the fluorescence of the gel of **1**. For the addition of Eu^3+^, the fluorescence intensity was decreased by approximately 80.62%. In contrast, the fluorescence of the gel of **1** was mostly quenched by Hg^2+^ with the same conditions. It was concluded that the gel of **1** could selectively detect Hg^2+^ via the simultaneous variation in the fluorescence and gel state.

**Figure 10:**
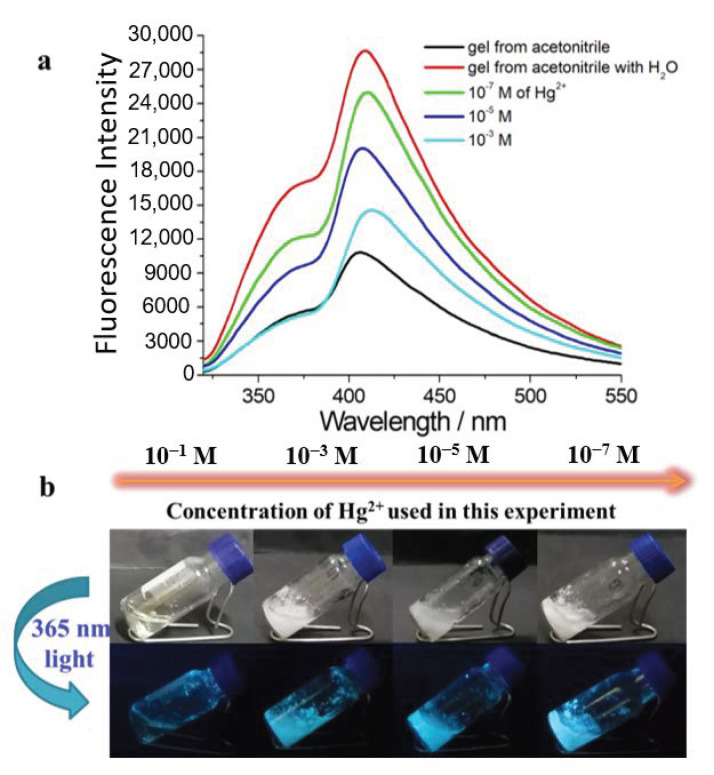
(**a**) Fluorescence emission spectra of gels of **1** after the addition of Hg^2+^ aqueous solution with the different concentrations; (**b**) the changes in gel state and emission light for gels of **1** after the addition of Hg^2+^ aqueous solution with the different concentrations. The addition amount of Hg^2+^ aqueous solution was 70 μL.

For investigating the detection ability of the gel of **1** in Hg^2+^, 70 μL of the Hg^2+^ aqueous solution with different concentrations was added to the gel of **1**. When Hg^2+^ was added to the gel of **1**, the gel state collapsed to a different extent, indicating that Hg^2+^ destroyed the noncovalent interactions of the gel of **1** in acetonitrile. The decrement in fluorescence emission intensity of the gel of **1** was gradually enlarged with the increase in Hg^2+^ concentration (Figure 10a). However, UV–vis absorption spectra of the gel of **1** with the addition of the Hg^2+^ aqueous solution with different concentrations did not significantly change, showing that the electron transition of π–π* of molecule **1** was not changed by Hg^2+^ (Figure S3). To further understand the interaction between gelator **1** and Hg^2+^, the morphology change of the gel of **1** with the addition of Hg^2+^ was observed (Figure S4). In Figure 10, Hg^2+^ destroyed the original self-assembly structure in the gel of **1**, and the nanofibers were changed to microspheres. The change in the self-assembly structure was possible because the coordination interaction between Hg^2+^ and the pyridine group in molecule **1** destroyed the intrinsic noncovalent interaction in the gel of **1**. The changes in the gel state and fluorescence emission of the gels of **1** after the addition of the Hg^2+^ aqueous solution with the different concentrations were made as images in Figure 10b. Gel states were changed to partial gel, and then, solutions of **1** with the different concentrations of Hg^2+^ aqueous solution increased from 10^−7^ to 10^−1^ M. Meanwhile, the fluorescence emission of gel was also gradually decreased in this experiment.

**Figure 11:**
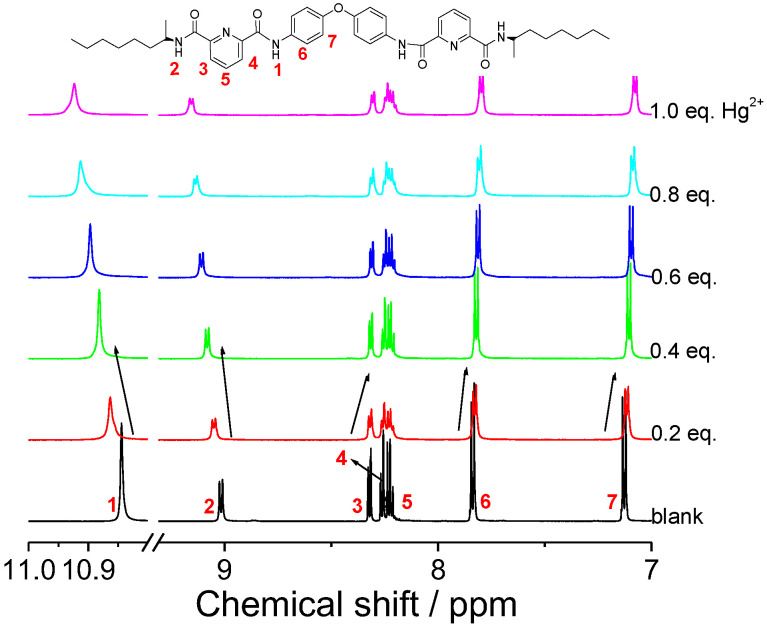
The partial ^1^H NMR titration experiments of molecule 1 by Hg^2+^ in DMSO-d_6_ at 298 K. The concentration of gelator **1** was 11.6 mM.

In order to study the coordination interaction between Hg^2+^ and the pyridine group in molecule **1** and the change in noncovalent interaction, NMR titration experiments with Hg^2+^ in DMSO-d_6_ (11.6 mM) were performed, as shown in Figure 11. Upon the gradual addition of Hg^2+^ to solution **1** in DMSO-d_6_, the chemical shifts of two active protons assigned to two amide groups (H1 and H2) were shifted downfield from 10.84 and 9.01 ppm to 10.92 and 9.15 ppm, respectively. The chemical shifts of H3–H7 of pyridine and benzene rings in molecule **1** were all moved upfield from 8.33, 8.27, 8.24, 7.85 and 7.14 ppm to 8.31, 8.25, 8.22, 7.79 and 7.07 ppm, respectively. These results indicated that hydrogen bonding in the gel system was destroyed by Hg^2+^, and the coordination interaction between molecule **1** and Hg^2+^ existed [72].

## 3. Conclusions

We designed a bis-pyridine-based gelator **1** with gelation abilities in six solvents, including some mixed solvents. Nanofiber and nanorod structures were formed in the gel systems. Xerogels of **1** from the above solvents exhibited hydrophobicity and hydrophilicity with the water contact angles of 141°–51°. Gelator **1** showed the obvious AIE behavior in its aggregate state. The gel of **1** in acetonitrile could respond to Hg^2+^ via the change in the fluorescence and gel state. Metal coordination interaction was possibly the main reason for inducing the fluorescence quenching and the damage in noncovalent interactions of the gel system. This study provides a new method for constructing a functional supramolecular self-assembly system as a sensor for the detection of Hg^2+^. 

## 4. Experimental Section

### 4.1. Reagents and Solvents

4-(4-aminophenoxy)benzenamine and (R)-octan-2-amine were purchased from Jiangsu Aikang Biomedical Research and Development Co. Ltd. Nanjing, China. 2,6-Pyridinedicarboxylic acid monomethyl ester was purchased from Zhengzhou Alpha Chemical Co. Ltd. All the reagents and solvents were analytically pure and without further purification. Water was deionized and obtained by triple distillation.

### 4.2. Techniques and Instrumentations

The techniques and instrumentations are shown in the supporting information.

## Figures and Tables

**Table 1:** Gelation performance of gelator **1**.

Solvents	1	Solvents	1
n-hexane	I	1, 4-dioxane	P
toluene	P	ethyl acetate	G(25)
methanol	G(25)	petroleum ether	I
ethanol	G(25)	DMSO/H_2_O = 4:1(*v*/*v*)	G(6.67)
acetonitrile	G(25)	DMF/H_2_O = 4:1(*v*/*v*)	G(20)
acetone	I	THF	S

P = precipitate; S = soluble; I = insoluble; G = gel. The values in parentheses are the critical gel concentration (CGC, mg mL^−1^).

## Data Availability

Not applicable.

## Supplementary Materials

The following supporting information can be downloaded at: https://www.mdpi.com/article/10.3390/gels8080464/s1, Techniques; Figure S1, XRD patterns of xerogels of **1** from different solvents and powder **1**; Figure S2, Images of solutions of **1** (10^−5^ M)in acetonitrile with different water content under 365 nm light.; Figure S3, UV-vis absorption spectra of gels **1** after the addition of Hg^2+^ with the different concentrations. The addition amount of Hg^2+^ aqueous solution was 70 μL.; Figure S4, FESEM image of acetonitrile gel **1** after the addition of Hg^2+^. of the experimental procedures, characterization data and some experimental results were provided in supporting information.
