# Supplementary material for: Bis-Pyridine-Based Organogel with AIE Effect and Sensing Performance towards Hg2+
Source: Gels. 2022 Jul 25;8(8):464. doi: 10.3390/gels8080464 (PMC9331886; doi:10.3390/gels8080464)
Supplement: Supplementary file 1 [file gels-08-00464-s001.zip › gels-1815834-supplementary.pdf]

gels

MDPI

Article

Bis-Pyridine-Based Organogel with AIE Effect and Sensing Performance towards Hg^{2+}

Aiping Gao, Qingqing Han, Qingqing Wang, Rong Wan *, Huijuan Wu and Xinhua Cao *

College of Chemistry and Chemical Engineering and Synthesis Key Laboratory of Xinyang City, Xinyang Normal University, Xinyang 464000, China; gaoapchem@163.com (A.G.); hqq9627@163.com (Q.H.); 18939697579@163.com (Q.W.); wuhjchem@163.com (H.W.)
* Correspondence: wanrong1992@163.com (R.W.); caoxhchem@163.com (X.C.)

Supplementary Materials

Citation: Gao, A.; Han, Q.; Wang, Q.; Wan, R.; Wu, H.; Cao, X. Bis-Pyridine-Based Organogel with AIE Effect and Sensing Performance towards Hg^{2+}. *Gels* **2022**, *8*, 464. https://doi.org/10.3390/gels8080464

Academic Editor(s): Jean-Michel Guenet

Received: 29 June 2022
Accepted: 21 July 2022
Published: 25 July 2022

Publisher's Note: MDPI stays neutral with regard to jurisdictional claims in published maps and institutional affiliations.

Copyright: © 2022 by the authors. Licensee MDPI, Basel, Switzerland. This article is an open access article distributed under the terms and conditions of the Creative Commons Attribution (CC BY) license (https://creativecommons.org/licenses/by/4.0/).

Techniques

^1H NMR and ^{13}C NMR spectrogram were recorded in DMSO-d_6 on a 600 MHz and 150 MHz nuclear magnetic resonance spectrometer from Japan Electronics Co., LTD. (JNM—ECZ600R/S3). High resolution mass spectrometer (HRMS) was recorded on a LTQ-Orbitrap mass spectrometer (Thermo Fisher Technology Co., LTD). FESEM images were obtained by SEM S4800 instrument (Hitachi, Ltd., Tokyo, Japan). Samples were prepared by dropping diluted gel on silicon wafer, freeze-drying and coating with Pt. The temperature and vacuum of the freeze-drying technology were at -55 ^{0}C and 10 Pa, respectively. X-ray Powder diffractions were performed by using a Philips PW3830 (Philips, Ltd., Eindhoven, Holland) with a power of 40 kV at 40 mA (Cu target, λ = 0.1542 nm). UV–vis absorption spectra were recorded on a UV–vis 3900 spectroscope (Hitachi, Ltd., Tokyo, Japan). Fluorescent emission spectra were obtained on Edinburgh Instruments FLS 1000 (Edinburgh Instruments, Ltd. Livingston, UK). Fourier transform infrared spectroscopy (FT-IR) was measured by Nexus 470 spectrometer (Thermo Fisher Scientific Co., LTD.). Water contact angles were performed using the sessile drop method (Dataphysics, OCA 20). The water droplets were introduced using a microsyringe, and images were captured to measure the angle of the liquid-solid interface. All samples were recorded at three different points.

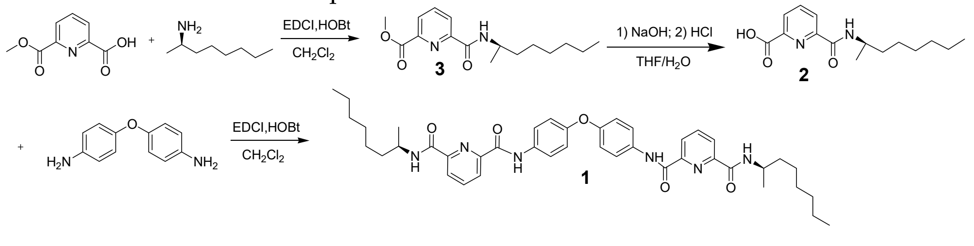

Scheme S1. The synthetic route of compound **1**.

Synthesis of compound **3**. 2, 6-Pyridinedicarboxylic acid monomethyl ester (1.00 g, 11.05 mmol), (R)-octan-2-amine (0.71 g, 11.05 mmol), (E)-3-(ethyldiazenyl)-N, N-dimethylpropan-1-amine hydrochloride (EDC) (2.12 g, 22.10 mmol), 1-Hydroxybenzotriazole hydrate (HOBt) (0.75 g, 11.05 mmol), mixed in CH_2Cl_2 (70ml) and stirred 12h at room temperature. Then the solvent was removed by vacuum distillation, and the residue was purified by silica gel column chromatography using petroleum/CH_2Cl_2 = 1/1 (v/v) as the eluent to afford compound 1 as a white powder.

Synthesis of compound **2**. Compound **3** (1.90 g, 6.50 mmol), NaOH (0.52 g, 13.00 mmol), added to 120ml THF/H_2O (1/1, v/v) and stirred 12h at room temperature. Afterwards, THF was removed under reduced pressure, the pH value was adjusted to 2-3

with dilute hydrochloric acid solution. Solid was precipitated and extracted, and white solid was obtained as compound **2**.

Synthesis of gelator **1**. The synthesis route of gelator **1** was similar to compound **3**, and white solid was obtained. ^{1}HNMR (600MHz, CDCl3) : 9.52 (s, 2H), 8.45 (d, J=6Hz, 2H), 8.43 (d, J=6Hz, 2H), 8.11 (t, J=12Hz, 2H), 7.7 (d, J=6Hz, 4H), 7.52 (d, J=6Hz, 2H), 7.06 (d, J=12Hz, 4H), 4.25 (m, 2H), 1.60-1.65 (m, 4H), 1.41 (m, 4H), 1.34 (m, 4H), 1.31 (m, 8H), 1.28 (d, J=6Hz, 6H), 0.87 (t, J=12Hz, 6H) ; ^{13}CNMR (150 MHz, CDCl3) : 162.7, 161.4, 154.2, 149.4, 148.7, 139.4, 132.7, 125.6, 125.2, 122.1, 119.4, 45.8, 37.1, 31.7, 26.2, 21.0, 14.2. HRMS: $C_{42}H_{52}N_6O_5$ [M+H]$^+$ calculated to be 720.3999, found: 720.3893.

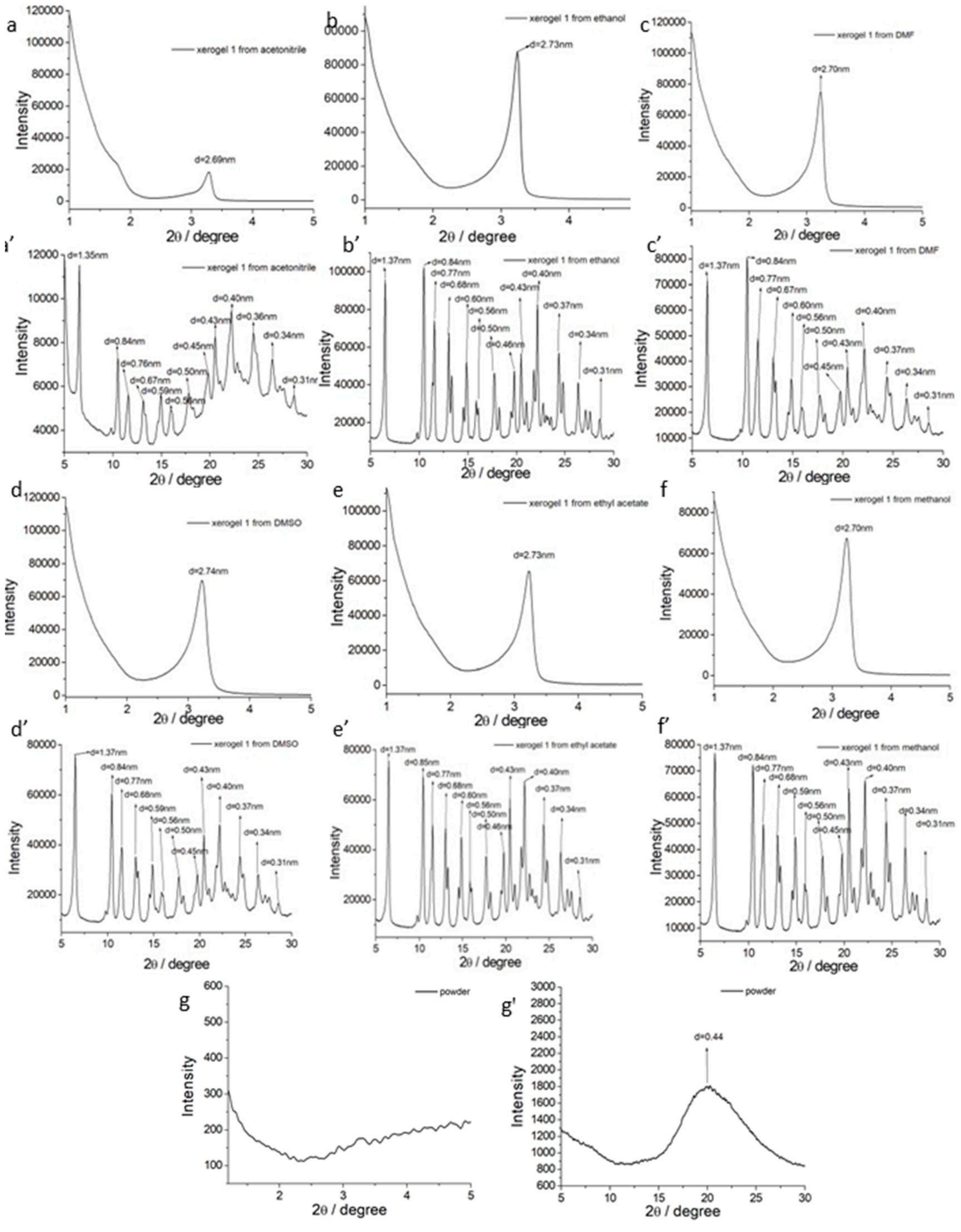

Figure S1. XRD patterns of xerogels of **1** from different solvents and powder **1**.

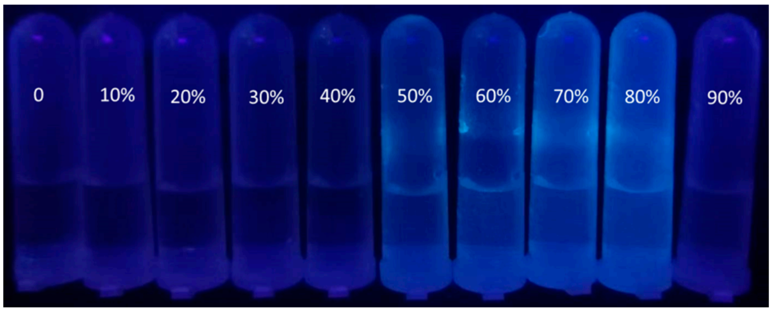

Figure S2. Images of solutions of **1** (10^{-5} M)in acetonitrile with different water content under 365 nm light.

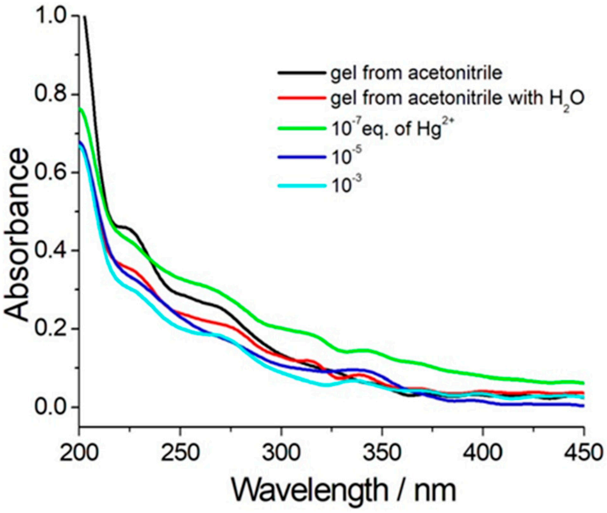

Figure S3. UV-vis absorption spectra of gels **1** after the addition of Hg^{2+} with the different concentrations. The addition amount of Hg^{2+} aqueous solution was 70 μL.

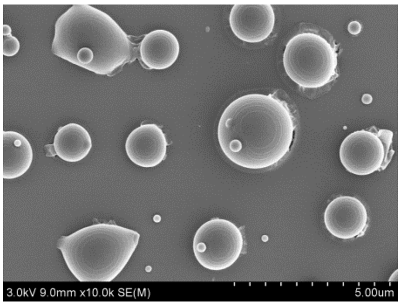

Figure S4. FESEM image of acetonitrile gel **1** after the addition of Hg^{2+}.
